# A unique double tango: Construct validation and reliability analysis of risk perception, attitude and practice (RPAP) questionnaire on dengue infection

**DOI:** 10.1371/journal.pone.0256636

**Published:** 2021-08-24

**Authors:** Mohd ‘Ammar Ihsan Ahmad Zamzuri, Mohd Nazrin Jamhari, Hasanain Faisal Ghazi, Muhamad Hazizi Muhamad Hasani, Noor Khalili Mohd Ali, Mohammad Faid Abd. Rashid, Rozita Hod, Rahmat Dapari, Mohd Rohaizat Hassan

**Affiliations:** 1 Department of Community Health, Universiti Kebangsaan Malaysia, Wilayah Persekutuan Kuala Lumpur, Malaysia; 2 Seremban District Health Office, Seremban, Negeri Sembilan, Malaysia; 3 State Department of Health Negeri Sembilan, Seremban, Negeri Sembilan, Malaysia; 4 State Department of Health Kedah, Alor Setar, Kedah, Malaysia; 5 College of Nursing, Al-Bayan University, Baghdad, Iraq; 6 Department of Community Health, Universiti Putra Malaysia, UPM Serdang, Seri Kembangan, Selangor, Malaysia; Management and Science University, MALAYSIA

## Abstract

**Introduction/background:**

Dengue fever remains a public health threat despite being preventable. A solution to the constant problem of dengue infection will require active intervention and a paradigm shift. Assessing perceived risk and correlating it with the attitude and practice of the community will help in designing appropriate measures. However, possible instruments for these assessments come with limitations.

**Objective:**

The aim is to develop and validate a new scoring-based questionnaire, using dual statistical approaches to measure risk perception, attitude, and practices (RPAP) related to dengue in the community.

**Methods:**

The RPAP questionnaire was developed bilingually using the International Society for Pharmacoeconomics and Outcome Research (ISPOR) guidelines. Content analysis was reviewed scrupulously by four expert panels. The initial 35-item scale was tested among 253 Malaysian respondents recruited non-probabilistically via multiple online platforms. Two statistical methods were employed to measure the construct validity: Exploratory Factor Analysis (EFA) as part of the Classical Test Theory (CTT) measurement, while Rasch Measurement Analysis (Rasch) was performed for the Item Response Theory (IRT) measurement. All results were cross-validated with their counterpart to ensure stability. Confirmatory Factor Analysis (CFA) was used to obtain a model fit index.

**Results:**

29 questions were retained after the final analysis. Both EFA and Rasch analysis detect multidimensionality. Nine latent factors were extracted from EFA, while only eight factors remained in the final model following CFA: 1) perceived susceptibility; 2) perceived severity; 3) perceived barrier; 4) perceived benefit; 5) cues to action; 6) self-efficacy; 7) attitude; and 8) practice. All items had adequate factor loadings and showed good internal consistency. The final model after CFA achieved a good fit with an RMSEA value of 0.061, SRMR of 0.068, PNFI of 0.649, and GFI of 0.996.

**Conclusion:**

The RPAP questionnaire contains 29 items and is a reliable and accurate psychometric instrument for measuring the risk perception of dengue fever, attitude, and practice of the community in dengue prevention. The Rasch measurement provides additional rigour to complement the CTT analysis. This RPAP questionnaire is suitable for use in studies related to dengue prevention in the community.

## Introduction

Dengue is a vector-borne disease that remains a significant public health problem. The World Health Organisation (WHO) estimated that nearly 390 million dengue infections occur per year with increasing mortality rates worldwide [1]. In Malaysia, the number of reported dengue cases has risen steadily ever since the disease’s discovery in 1902 [2, 3]. Without effective and safe vaccines, prevention is heavily dependent on vector control targeting the *Aedes* sp. mosquito.

Entomological accounts of *Aedes* sp. explain its ability to effectively transmit dengue virus (DENV), from the ability of the egg to survive a lengthy drought [4, 5] to the transovarial mode of DENV transmission [6, 7]. This vector naturally breeds in human habitat, unlike vectors for other diseases such as malaria. Additionally, a local epidemiological study has elucidated the socio-ecological factors that contribute to ongoing disease transmission. Non-modifying ecological factors, such as temperature, rainfall, and humidity, have been shown to affect disease transmission [8]. Increasing population and rapid urbanisation have rapidly and dramatically changed the ecological landscape. At the individual level, human activities on daily basis may indirectly create breeding grounds for the *Aedes* sp. Examples of unintended breeding promotion are improper disposal of solid waste, unplanned landfills, improper storage of water container, or clogged water flow [9, 10]. In addition, low levels of participation in community-based dengue prevention programmes, such as Communication for Behavioural Impact (COMBI) [11], also contributed to worsening the problem [3].

Attitudes and practices towards dengue prevention need to be evaluated to ensure the efficacy of health education and information disseminated to the public. Moreover, an accurate measurement of the perceived risk of dengue infection is necessary to correlate with attitude and practice [12]. Risk perception is defined as a multidimensional assessment by an individual of the likelihood of having an unpleasant experience [13]. In order to avoid harm, a person must have a positive attitude and reasonable practices for prevention, as per the Health Belief Model (HBM) theory. This theory proposes six factors that shape a person’s behaviour: perceived susceptibility, perceived vulnerability, perceived benefit, perceived barrier to practice, cues to take action, and self-efficacy.

If measuring either risk perception, attitude, or practice is already a challenge, it is even more difficult to measure all three elements together. Administering a valid and reliable psychometric scale is of the utmost necessity. Despite the availability of published local literature regarding dengue knowledge, attitude, and practice (KAP), a slight ambiguity was found during the assessment of the questionnaire or tool used for the measurement in this work. The majority reported the value of internal consistency of their tools, while the validity measures were less likely to be mentioned. For this reason, a sufficient and thoroughly validated questionnaire is necessary for future research.

This study aims to develop and validate a risk perception, attitude, and practice (RPAP questionnaire) for dengue infection that can be used in any community suffering from the disease. This study attempts to further ensure the reliability of the questionnaire through a unique dual approach, featuring both Classical Test Theory (CTT) and Item Response Theory (IRT).

## Methods

### The questionnaire development process

The RPAP questionnaire is a newly developed instrument to measure risk perception, attitude, and community practice related to dengue. It was designed as a self-administered scored survey, and bilingual in both English and Malay. Items were initially developed in English and the translation process followed guidelines by the International Society for Pharmacoeconomics and Outcome Research (ISPOR) [14]. The translation process involved two linguists, two Public Health Physicians, and two academicians, to ensure that the result was accurate, rigorous, and followed good practices in cultural adaption [15]. The steps used to develop the scale were based on DeVellis’ protocol [16]:

1. *Construct development*: The development of the constructs was based on a previous study on a socio-ecological framework of prolonged dengue outbreak in the community. The work is intended to measure three attributes, namely, risk perception, attitude, and practice.
2. *Item pool development*: The items were formulated in reference to the ‘Standard Questionnaire On Risk Perception Of An Infectious Disease Outbreak’ tool [17] and the application of established human behavioural theory, which is the Health Belief Model (HBM). Two postgraduate DrPH students and two academicians from Universiti Kebangsaan Malaysia (UKM) performed this process systematically. The preliminary set of questionnaires consisted of 43 items constructed during this step.
3. *Format ascertainment*: The instrument was designed to be scored based on an 8-point Likert scale, with a score of 1 = strongly disagree and a score of 8 = strongly agree. No neutral (middle) score was assigned, as the researchers intended to assess the potential selection of the respondents.
4. *Item pool review*: All developed items were reassessed individually to ensure their fitness for measuring the necessary constructs. Each item was rated once by three experts (one academician from the Universiti Putra Malaysia and three field epidemiologists) who decided whether to omit or include each item. An item that was approved by at least three experts was included in the final consensus. Only 35 items remained in the final version of the RPAP questionnaire, with 12 items representing risk perception, 10 items for attitude, and 12 items for practice.
5. *Pretesting*: The RPAP questionnaire was pretested on ten volunteers to check language understanding among the laymen before conducting the validation study. Some minor adjustments were made, especially regarding the complexity of the constructed sentences.

### Study design and settings

The population-based cross-sectional study for validating the RPAP questionnaire was conducted over two weeks in December 2020 during the third wave of the COVID-19 pandemic in Malaysia. Due to the enforcement of a second Movement Control Order (MCO) at the national level, the country on a strict lockdown. The respondents were therefore recruited via an online open-source website, email blasts, and Whatsapp. Each of these modalities linked to a Google form. The choice to use a digital platform was based on two important points; 1) the risk of an in-person survey due to droplet transmission of COVID-19 virus, and 2) the large proportion of the Malaysian population with easy access to digital media [18]. The web-based questionnaire was set to only allow access after the subject had read and responded to the terms and conditions. The common sample size used in validation studies ranges between 5 and 10 respondents per item [19]. The targeted range of respondents in this study was therefore between 175 and 350 samples.

### Statistical analysis

The RPAP questionnaire was subjected to double statistical modalities to measure its construct validity. The commonly used method, Classical Test Theory (CTT), was performed using JASP software, while Item Response Theory (IRT) via Rasch Measurement Model Analysis was performed using Bond & Fox Steps software. Both software are free and accessible to the public. Rasch analysis has several advantages in interpreting attitudinal scale [20], as it can simultaneously provide a comprehensive result for latent traits measured [21].

All 35 items were computed in the analysis. For the Exploratory Factor Analysis (EFA) measurement, factor extraction was performed using Principal Component Analysis (PCA), while the promax oblique rotation method was used. The total number of factors retained was determined using the Kaiser criterion with eigenvalues greater than one. Items were suppressed when the factor loading was less than 0.35. The Kaiser-Meyer-Olkin (KMO) index was employed for sample adequacy with Bartlett’s test for sphericity valuation. Reliability analysis was performed, and the value of Cronbach’s alpha and McDonald’s omega were measured.

### Ethical approval

This study received ethical approval from the Universiti Kebangsaan Malaysia (UKM) Research Ethics Committee **(UKM PPI/111/8/JEP-2019-854)** and the Medical Research & Ethics Committee of the Ministry of Health Malaysia (**NMRR-19-3909-51875)** and was registered with National Medical Research Register (NMRR). Respondents gave their consent prior to participation in this study.

## Results

### Background of respondents

Based on Table 1, the mean age of the respondents from the online survey was 33.6 (SD 6.2). The majority were female, ethnically Malay, held a degree in educational achievement, worked as a civil servant, and owned a landed terrace property. More than three-quarters of the respondents had no personal history of dengue infection. However, 51.8% of the respondents had relatives that had at one point contracted the dengue fever. Table 2 presents all the items in the newly developed RPAP questionnaire, with mean marks of the individual questions and the construct.

**Table 1:** Characteristics of online respondents in the study.

	N (Total = 253)	Percentage (%)
Age		
≤ 20 years	7	2.8
21–25 years	12	4.7
26–30 years	29	11.5
31–35 years	135	53.4
36–40 years	48	19.0
41–45 years	11	4.2
46–50 years	6	2.4
> 50 years	5	2.0
Mean age (SD)	33.6 (± 6.2)	
Gender		
Male	106	41.9
Female	147	58.1
Ethnicity		
Malay	216	85.4
Chinese	16	6.3
Indian	13	5.1
Others	8	3.2
Educational Attainment		
At least secondary school	10	4.0
Diploma/STPM	38	15.0
Degree	141	55.7
Master	62	24.5
PhD	2	0.8
Occupation		
Civil servant	157	62.0
Private sector	55	21.7
Self-employed	7	2.8
Not working	31	12.3
Retired	3	1.2
Residential type		
Landed bungalow	20	7.9
Landed terrace	152	60.1
High rise	50	19.8
Traditional house	27	10.6
Others	4	1.6
Personal dengue history		
Yes	43	17.0
No	210	83.0
Dengue history among family/relative		
Yes	131	51.8
No	122	48.2

**Table 2:** Details of the items, constructs and the corresponding questions’ mean marks of each question, construct, and overall score (N = 253) based on the initial questionnaire development categories of risk perception, attitude, and practice.

No	Code^a^	Construct and Questions^b^	Mean (± SD)
Risk Perception (Total mark: 94)^c^			
Objective: To measure the perceived threat of dengue infection.			
(*Scale*: *1 strongly disagree–8 strongly agree)*			
1	D1	I am at risk to get dengue fever	4.77 (2.05)
2	D2	Dengue fever is a seasonal disease, I will be safe from it if the dengue season has passed.	2.60 (1.99)
3	D3	I am bitten by mosquitoes every day, but I have never been infected with dengue fever. So I am not at risk of getting dengue fever.	2.18 (1.69)
4	D4	Dengue fever can cause death.	7.87 (0.58)
5	D5	Fever for 3 days is worrisome to me. I feel that I cannot wait up to 5 days to get treatment.	7.26 (1.45)
6	D6	I have many close friends who have recovered from dengue fever, but I am still afraid of dengue.	5.87 (2.08)
7	D7	I need to be involved in every health campaign aimed to destroy mosquito breeding place, as it helps reduce the risk of dengue to my family.	6.63 (1.51)
8	D8	With at least one person who is knowledgeable about the disease in the house, he/she can help prevent the disease in the home.	7.34 (1.29)
9	D9	All the time and money I spent to stop dengue is worthwhile because I’m concerned about living a healthier lifestyle.	6.69 (1.50)
10	D10	I need a lot of money to implement dengue prevention at home.	2.25 (1.56)
11	D11	I am very busy until I have no time to implement dengue prevention at home.	3.30 (1.91)
12	D12	I need to spend the weekend with my family rather than participating in gotong royong to prevent dengue.	4.56 (2.27)
Attitude (Total mark: 80)^b^			
Objective: To measure attitude towards dengue prevention.			
(*Scale*: *1 strongly disagree–8 strongly agree)*			
13	E1	It is necessary for me to take precautions if my area is declared an outbreak (WTK/hotspot) area.	7.68 (0.76)
14	E2	I become interested to take part in control/prevention of dengue when a construction site in the neighbourhood was suspended for Aedes breeding spot.	6.13 (1.74)
15	E3	It is necessary for me to ensure old and unused items that can store water, are kept closed.	7.84 (0.50)
16	E4	Abandoned and damaged vehicles in the neighbourhood trigger my intention to take the necessary action.	6.32 (1.75)
17	E5	It is necessary to ensure no “illegal structures” in the neighbourhood are left unattended.	7.31 (1.30)
18	E6	It is necessary for me to ensure there are no breeding spots around my house.	7.44 (1.21)
19	E7	It is necessary for me to deliver information about dengue fever to my family members.	7.58 (0.75)
20	E8	It is necessary for me to ensure that there are no illegal dumping sites in my neighbourhood.	7.21(1.18)
21	E9	It is necessary for me to ensure that the drainage or water flow system in my house to be properly maintained.	7.58 (0.74)
22	E10	I become more interested to take part in control/prevention of dengue when there are cooperation within the neighbourhoods.	7.12 (1.16)
Practice (Total mark: 104)^b^			
Objective: To measure actual action taken by the respondent to prevent dengue infection			
(*Scale*: *1 strongly disagree–8 strongly agree)*			
23	F1	I use mosquito repellent (lotion/spray/coil).	6.74 (1.69)
24	F2	I always keep water containers in my house tightly closed.	7.19 (1.22)
25	F3	I check for potential mosquito breeding inside the house.	7.13 (1.30)
26	F4	I put larvicide into the water storage to kill the mosquito larvae.	5.29 (2.34)
27	F5	I only dispose rubbish at the designated place.	7.77 (0.66)
28	F6	I made complaint to the authority when I found an illegal dumping site.	6.20 (1.79)
29	F7	I keep my drainage system properly maintained.	7.23 (1.17)
30	F8	I do not keep unused items that can store water.	6.92 (1.48)
31	F9	I made complaint to the authority when there is damaged vehicle idling in my neighbourhood.	5.93 (1.90)
32	F10	I check for potential mosquito breeding place around the neighbourhood.	5.68 (2.09)
33	F11	I participate in gotong royong activities to prevent dengue.	6.51 (1.56)
34	F12	I made complaint to the authority when I found illegal garden.	5.49 (2.02)
35	F13	I made complaint to the authority when I found illegal building structure.	5.97 (1.82)

^a^ All the codes were based on initial phase of development. Following Confirmatory Factor Analysis (CFA), the code might scattered based on latent traits.

^b^ Wording of the items in the table might differ from the actual way it was asked in the questionnaire, but the intended meanings are preserved.

^c^ It is the maximum score possible.

### Classical test theory using exploratory factor analysis (EFA)

The Kaiser-Meyer-Olkin (KMO) index for the RPAP-questionnaire was 0.879, whereas the p-value for Bartlett’s test of sphericity was significant at p < 0.001. The approximated chi-square and degree of freedom of the Bartlett’s test were 3816 and 595, respectively. This indicates that the sample size employed in the validation study was adequate to run a factor analysis. Reliability analysis on all 35 items from all constructs yielded an adequate measure of item-total correlation (ITC), with the value of Cronbach’s alpha between 0.415–0.908, and the value of McDonald’s omega between 0.447–0.912.

Two items were rejected via EFA, both from the attitude domain (E5 and E8) due to low factor loading (< 0.35). Some items were reassigned into new latent factors. Nine latent factors were detected from EFA despite the questionnaire’s design involving three domains. Domain practice consisted of two factors (Factor 1 and Factor 2), domain attitude consists of two latent factors (Factor 3 and Factor 4), and domain risk perception was comprised of the remaining five latent factors. Table 3 shows the items and their latent factors. The mean score for all the items ranged from 2.18 (item D3) to 7.87 (item D4).

**Table 3:** Results of EFA and reliability analysis of all 35 items with respective factor loadings, ITC, and domain-specific Cronbach α, as well as McDonald’s omega.

Domain	EFA									Reliability Analysis		
	Factor Loading into Extracted Factors									ITC	Cα	ω
Item Code	Factor 1	Factor 2	Factor 3	Factor 4	Factor 5	Factor 6	Factor 7	Factor 8	Factor 9			
Practice												
E4	0.906									0.768	0.908	0.912
E8	***0*.*349***									0.615		
F6	0.956									0.816		
F9	1.047									0.800		
F10	0.465									0.641		
F11	0.449									0.681		
F12	0.819									0.608		
F13	1.035									0.771		
F4		*0*.*330*								0.539	0.764	0.781
D7		0.409								0.605		
E1		0.830								0.419		
E2		0.484								0.575		
F1		*0*.*339*								0.378		
F2		0.611								0.505		
F7		0.536								0.554		
Attitude												
D8			0.897							0.314	0.586	0.593
E5			*0*.*348*							0.308		
E7			0.465							0.496		
E10			0.605							0.444	0.571	0.580
E3				0.766						0.454		
E9				0.353						0.403		
F5				0.773						0.463		
F8				0.400						0.385		
Risk Perception												
D9					0.428					0.486	0.755	0.768
E6					1.136					0.603		
F3					0.917					0.692		
D2						0.868						
D3						0.852						
D1						*0*.*189*			0.782			
D6								*0*.*079*	0.731			
D4								0.776				
D5								0.610				
D10							0.660			0.354	0.415	0.447
D11							0.736			0.294		
D12							0.481			0.142		

Abbreviations: EFA = exploratory factor analysis; ITC = item-total correlation; Cα = Cronbach’s alpha; ω = McDonald’s omega.

* Bold text denotes that the item was removed from being included in the CFA.

** Italic text denotes factor loading less than 0.35.

*** Underlines signify shared cross loading.

**** Reliability analysis of the underline items were according to the latent factor.

Cronbach’s alpha & McDonald’s omega can only be computed with minimum of 3 items. Hence not calculated for Factor 8 & Factor 9 that only have 2 items each.

### Confirmatory factor analysis (CFA)

**Fig 1:**
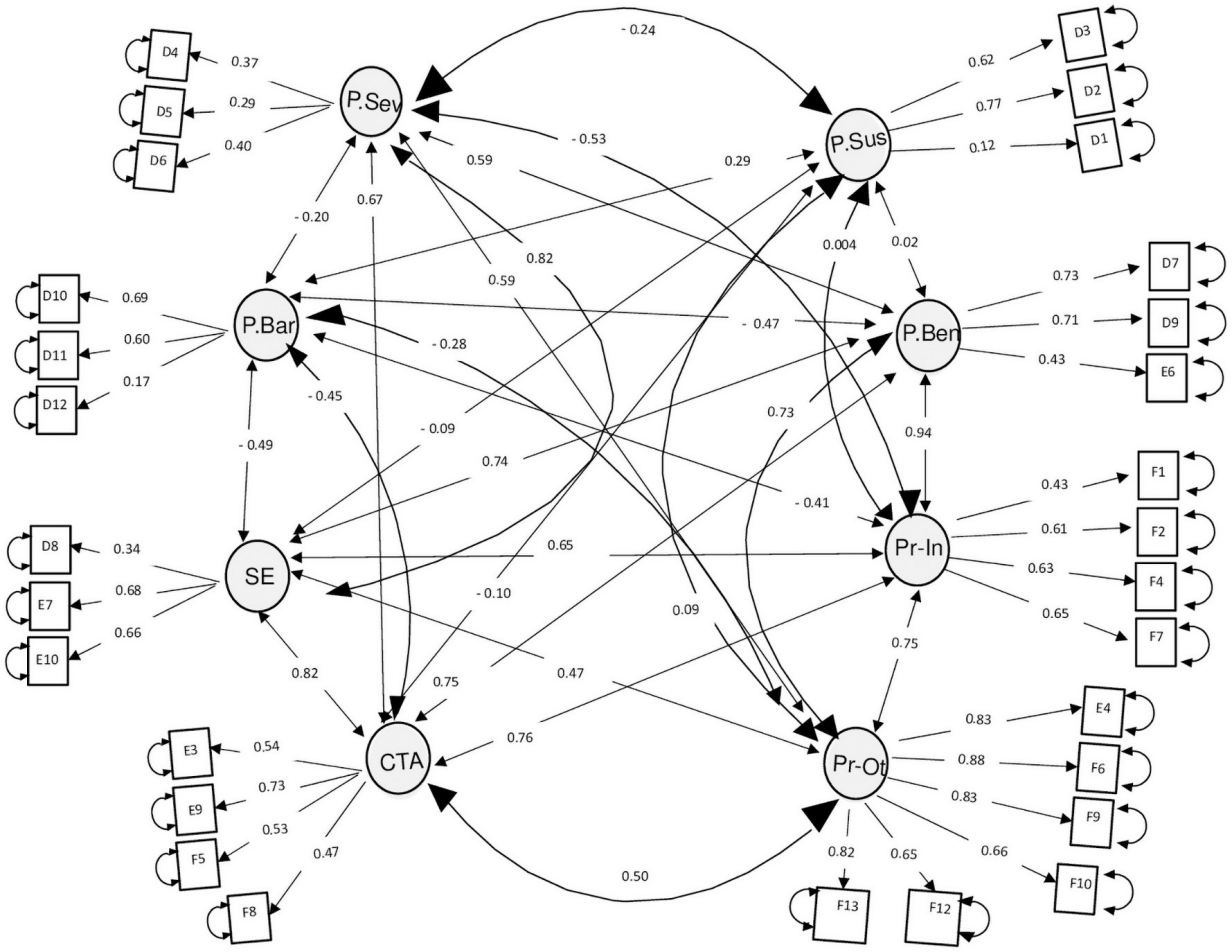
Path analysis of the confirmatory factor analysis (CFA) showing standardised estimates of the correlations (figures on the arrows) between the five domains (ellipse), the 31 items (rectangle). (P.Sev = Perceived Severity; P.Bar = Perceived Barrier; SE = Self-Efficacy; CTA = Cues-to-action; P.Sus = Perceived Susceptibility; P.Ben = Perceived Benefit; Pr-In = Practice Inside the housing compoung; Pr-Ot = Practice Outside the house compound namely the neighbourhood).

The analysis was conducted using the same dataset from the EFA to provide a better visualisation of the model. Although 33 items were included in the measurement study, only 29 items remained: four items (E1, E2, F3, and F11) were removed during analysis. The path analysis, Fig 1, showed that one latent factor (Factor 9) had collapsed, and the items had merged with another construct. Therefore, the final model consisted of eight domains; this new model showed a good model fit. The χ^2^ factor model was significant (p < 0.001), and the relative CMIN/DF is 1.96 (< 2.0), which indicates a good fit. According to standard practice, a combination of several fitness indices is more meaningful in interpreting the model fitness. From Table 4, the RMSEA value was 0.061, with an SRMR of 0.068, GFI of 0.996, CFI of 0.86, and PNFI of 0.649; these values achieved the desired target (a good fit). The calculated average variance extracted (AVE), maximum shared variance (MSV), and composite reliability were satisfactory. The standardised factor loading for all items and the correlation coefficient value for each latent factor are shown in Fig 1. Overall reliability analysis for the 29 items produced a Cronbach’s alpha value of 0.824 and a McDonald’s omega value of 0.841, indicating good consistency (cut off > 0.7).

**Table 4:** Result of MSV, AVE, CR, and fitness of the model obtained from CFA.

Latent Factor	Maximum Shared Variance	Average Variance Extracted	Composite Reliability
	(MSV)	(AVE)	(CR)
Practice (outside)	0.561	0.615	0.904
Practice (inside)	0.876	0.343	0.671
Perceived Benefit	0.876	0.409	0.665
Perceived Susceptibility	0.084	0.340	0.561
Perceived Severity	0.666	0.127	0.301
Perceived Barrier	0.237	0.287	0.500
Self-Efficacy	0.674	0.341	0.591
Cues to Take Action	0.674	0.333	0.660
Fit Indices		Value	Cut-off
Root mean square error of approximation (RMSEA)		0.061	< 0.08
Standardised root mean square residual (SRMR)		0.068	< 0.08
Goodness of fit index (GFI)		0.996	> 0.9
Parsimony Normed Fit Index (PNFI)		0.649	> 0.5
Comparative Fit Index (CFI)		0.860	> 0.9

### Item response theory using Rasch measurement analysis

This approach was used to cross-validate the EFA result. As shown in Table 5, the initial 35-item RPAP questionnaire has multidimensionality (value of > 2.0 for the unexplained variance of the contrast). All 35 items’ reliability and person reliability were good (> 0.7). A few items were found to be erratic but remained in the final model after a consensus review on the need for these items to provide or contribute to meaningful analysis.

**Table 5:** Result from item response theory (IRT).

No	Rasch Psychometric Measure	Result	Suggested cut-off
All 35 items measured			
1.	Dimensionality check		
	- Raw variance explained by measure	36.4%	40.0%
	- Unexplained variance in 1^st^ contrast	3.1	< 2.0
	- Unexplained variance in 2^nd^ contrast	2.3	< 2.0
2.	Item reliability	0.98	> 0.7
	Item separation index	6.83	> 2
	Person reliability	0.78	> 0.7
	Person separation index	1.88	> 2
3.	Response format, *Andrich Threshold logit*	All < 1.4 Refer Fig 2	1.4 < x < 5.0 Merge rating if < 1.4
Factor: Practice–Outside (7 items)			
1.	Uni-dimensionality check		
	- Raw variance explained by measure	31.6%	40.0%
	- Unexplained variance in 1^st^ contrast	1.7	< 2.0
2.	Item Reliability	0.95	> 0.7
3.	Fits statistic (range)		
	a) InFit		
	✓ MNSQ	0.69–1.49	0.5 < y < 1.5
	✓ Z std	–2.6 (F9)–4.6	–2 < z < 2
	b) OutFit		
	✓ MNSQ	0.66–1.55 (F10)	0.5 < y < 1.5
	✓ Z std	–3.5–4.8	–2 < z < 2
	c) Point-measure correlation	0.68–0.79	0.32 < r < 0.8
Factor: Practice–Inside (4 items)			
1.	Uni-dimensionality check		
	- Raw variance explained by measure	34.4%	40.0%
	- Unexplained variance in 1^st^ contrast	1.6	< 2.0
2.	Item Reliability	0.99	> 0.7
3.	Fits statistic (range)		
	a) InFit		
	✓ MNSQ	0.85–1.28	0.5 < y < 1.5
	✓ Z std	–1.2–2.4 (F1)	–2 < z < 2
	b) OutFit		
	✓ MNSQ	0.86–1.35	0.5 < y < 1.5
	✓ Z std	–1.5–2.6	–2 < z < 2
	c) Point-measure correlation	0.56–0.81 (F4)	0.32 < r < 0.8
Factor: Cues to Action (4 items)			
1.	Uni-dimensionality check		
	- Raw variance explained by measure	43.2%	40.0%
	- Unexplained variance in 1^st^ contrast	1.9	< 2.0
2.	Item Reliability	0.98	> 0.7
3.	Fits statistic (range)		
	a) InFit		
	✓ MNSQ	1.05–1.24	0.5 < y < 1.5
	✓ Z std	0.3–1.4	–2 < z < 2
	b) OutFit		
	✓ MNSQ	0.78–1.11	0.5 < y < 1.5
	✓ Z std	–0.9–0.8	–2 < z < 2
	c) Point-measure correlation	0.50–0.84	0.32 < r < 0.8
Factor: Self-Efficacy (3 items)			
1.	Uni-dimensionality check		
	- Raw variance explained by measure	21.9%	40.0%
	- Unexplained variance in 1^st^ contrast	1.7	< 2.0
2.	Item Reliability	0.94	> 0.7
3.	Fits statistic (range)		
	a) InFit		
	✓ MNSQ	0.80–1.42	0.5 < y < 1.5
	✓ Z std	–1.4–2.4 (D8)	–2 < z < 2
	b) OutFit		
	✓ MNSQ	0.82–1.12	0.5 < y < 1.5
	✓ Z std	–1.4–0.9	–2 < z < 2
	c) Point-measure correlation	0.64–0.79	0.32 < r < 0.8
Factor: Perceived Susceptibility (3 items)			
1.	Uni-dimensionality check		
	- Raw variance explained by measure	43.4%	40.0%
	- Unexplained variance in 1^st^ contrast	1.8	< 2.0
2.	Item Reliability	0.99	> 0.7
3.	Fits statistic (range)		
	a) InFit		
	✓ MNSQ	0.98–1.04	0.5 < y < 1.5
	✓ Z std	–0.1–1.1	–2 < z < 2
	b) OutFit		
	✓ MNSQ	0.90–1.2	0.5 < y < 1.5
	✓ Z std	–0.9–2.1 (D1)	–2 < z < 2
	c) Point-measure correlation	0.63–0.68	0.32 < r < 0.8
Factor: Perceived Severity (3 items)			
1.	Uni-dimensionality check		
	- Raw variance explained by measure	35.1%	40.0%
	- Unexplained variance in 1^st^ contrast	1.7	< 2.0
2.	Item Reliability	0.99	> 0.7
3.	Fits statistic (range)		
	a) InFit		
	✓ MNSQ	0.74–1.55 (D4)	0.5 < y < 1.5
	✓ Z std	–2.9–3.3	–2 < z < 2
	b) OutFit		
	✓ MNSQ	0.90–1.26	0.5 < y < 1.5
	✓ Z std	–0.2–1.6	–2 < z < 2
	c) Point-measure correlation	0.31–0.81	0.32 < r < 0.8
Factor: Perceived Barrier (3 items)			
1.	Uni-dimensionality check		
	- Raw variance explained by measure	43.7%	40.0%
	- Unexplained variance in 1^st^ contrast	1.9	< 2.0
2.	Item Reliability	0.99	> 0.7
3.	Fits statistic (range)		
	a) InFit		
	✓ MNSQ	0.91–1.19	0.5 < y < 1.5
	✓ Z std	–1.0–2.1	–2 < z < 2
	b) OutFit		
	✓ MNSQ	0.89–1.12	0.5 < y < 1.5
	✓ Z std	–1.0–1.3	–2 < z < 2
	c) Point-measure correlation	0.58–0.68	0.32 < r < 0.8
Factor: Perceived Benefit (3 items)			
1.	Uni-dimensionality check		
	- Raw variance explained by measure	25.7%	40.0%
	- Unexplained variance in 1^st^ contrast	1.6	< 2.0
2.	Item Reliability	0.98	> 0.7
3.	Fits statistic (range)		
	a) InFit		
	✓ MNSQ	0.93–1.72	0.5 < y < 1.5
	✓ Z std	–0.6–4.5	–2 < z < 2
	b) OutFit		
	✓ MNSQ	0.86–1.18	0.5 < y < 1.5
	✓ Z std	–1.3–1.2	–2 < z < 2
	c) Point-measure correlation	0.57–0.79	0.32 < r < 0.8

**Fig 2:**
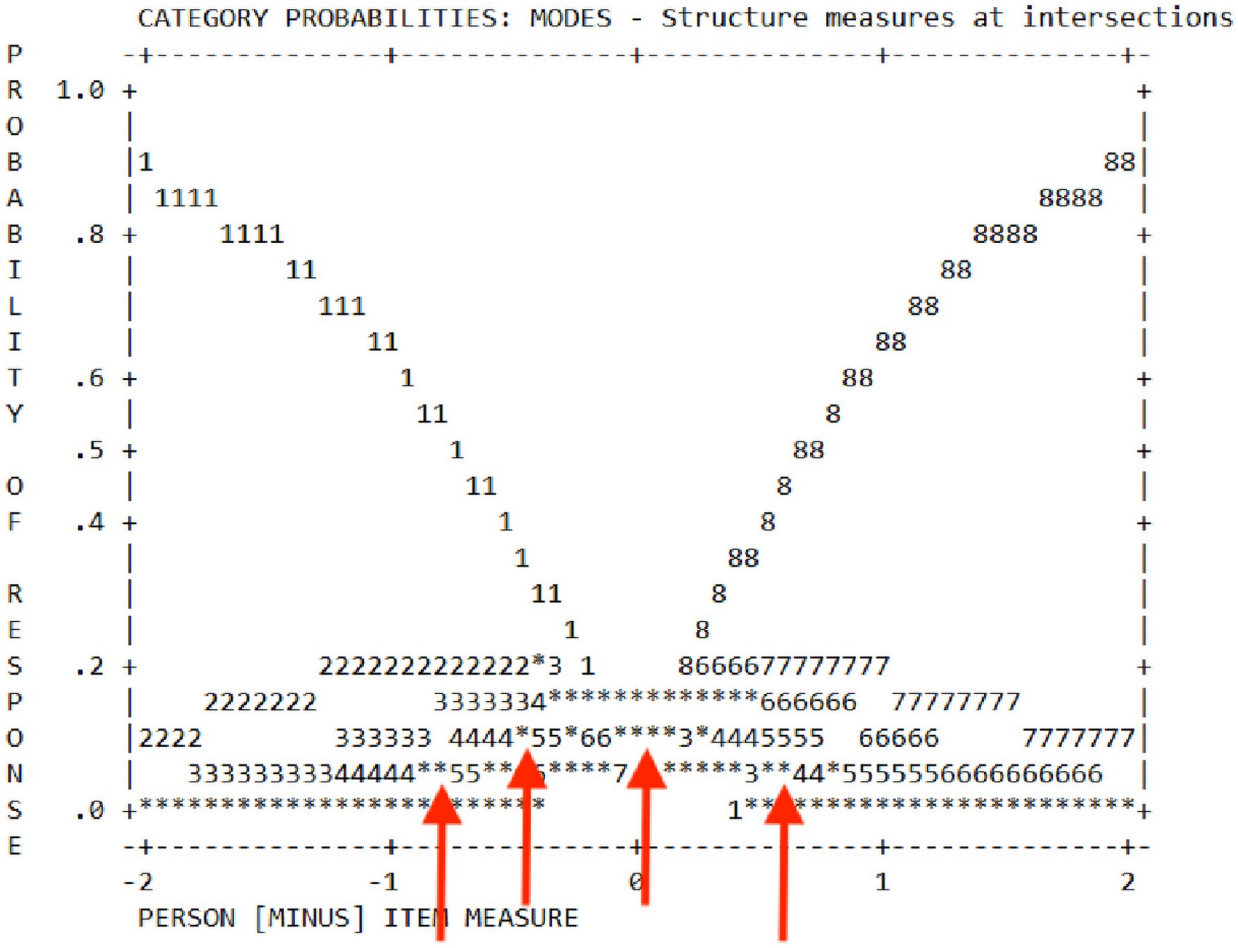
Probability curves for Likert scale.

An assessment using the Likert scale suggested the merging of scales to achieve a better response (four instead of eight on the Likert scale), as evidenced by low *Andrich Threshold logit*, obtained 0.29 (1.4 < x < 5.0) and depicted by four-point arrows in the Probability Curve (Fig 2).

The overall percentage of raw variance explained by measures was 64.2% (> 60%). However, the residual contrast analysis showed elements of multidimensionality. In view of this violation of unidirectionality, the items were analysed according to the latent traits obtained from the EFA result. Almost all constructs showed a good and satisfactory result for InFit MNSQ, InFit Z standard, OutFit MNSQ, OutFit Z standard, item reliability, and point measure correlation, as demonstrated in Table 5. Hence, the dual approaches demonstrated similarly good psychometric properties.

## Discussion

Researchers are widely adopting questionnaire-based methods in the field of behavioural study due to their practicality, low cost, and convenience for large data collection. The present study has demonstrated that the psychometric performance of RPAP is sufficient across both CTT and IRT analyses, supporting the conclusion that the 29-item scale covering risk perception, attitude, and practice towards dengue infection is reliable and psychometrically valid. A self-reported instrument must be able to function appropriately across heterogeneity of population, be straightforward to be administered and scored, and follow fundamental human behavioural theory. Validation testing using factor analysis (FA) has become more prominent due to the quantitative nature of the measurement that produces objective parameters for comparison [22–24].

The two types of FA function differently. EFA is usually used for factor reduction, combining items into their common factors based on the pattern-linearity of the factor loadings [25]. Conversely, CFA harmonises the model obtained from EFA by verifying the discriminant and convergent validity (construct validity) of the extracted factors and their respective items [16]. In this study, the EFA analysis was carried out using the Principal Component Analysis (PCA) as its extraction method; however, this differs with the rotation method. Few studies employing the extraction method have produced valid results that adequately measure the risk perception and KAP domains [22, 23]. The oblique (promax) rotation was selected because of the theoretical assumption that the factors may have some correlation with each other rather than being mutually exclusive [26]. Conventional varimax rotation makes no correlated assumption among the factors, and would therefore have been less logical in this situation. Human behaviour (for example, motivation to participate in prevention programme) is very dynamic and shaped by many elements [27, 28].

The use of the Kaiser criterion to retain several factors led to the detection of nine latent factors extracted in EFA. This multidimensionality of the initial 35 items was similarly detected and proved via Rasch analysis. The Rasch method provided a different dimension reduction analysis by examining the residual components. A higher percentage of the first contrast of the residual (more than 2.0) might signify the existence of another dimension. Hence, this unique combination of measurements provided a good assessment of construct validity and reliability in complementing bidirectional relations [29].

The model derived from CFA was a good fit, as it suggested an acceptable discriminant and convergent validity. The use of multiple fitness indices has been widely adopted by scholars. The model χ^2^ result was highly sensitive to a number of observed variables or sample size [30]. The CFA was able to demonstrate good internal consistencies for all measured domains in the RPAP questionnaire, as evidenced by the acceptable range of composite reliability that is comparable with the regularly calculated Cronbach’s alpha. These internal consistencies were similar to the items’ reliability, as measured by the Rasch analysis, for each domain. Nonetheless, this paper takes a new perspective when reporting other types of reliability alongside Cronbach’s alpha, which is used extensively in about 90% of the literature [31]. Although the difference between the results is essentially minuscule, it is worth reporting for both coefficients, as the model used in this work could not be ascertained to have had a tau-equivalent reliability or to be a congeneric model [32–34].

### Congruency with latent factors

It is worth describing the eight measured latent factors from the initial three domains. Six factors in this questionnaire are thematically congruent with the Health Belief Model (HBM). The HBM is a well-established human behavioural model developed by Godfery Hochbaum, Stephan Kegels, and Irwin Rosenstock during the 1950s. The theory is highly recognised as an effective method of creating behavioural changes. It has been continuously evaluated and rejuvenated over time; nevertheless, its core components are still applicable today [35]. Measuring risk perception is essential to understand the population in the context of dengue infection and transmission before embarking on health education efforts. This research has demonstrated that the components of perceived risk in this study were made of all four HBM attributes, namely, perceived susceptibility, perceived severity, perceived benefit, and perceived barriers; while the attitude components represent the remaining domains, which are cues to action and self-efficacy.

Perceived susceptibility is a subjective perception of the threat of acquiring an illness and may vary between individuals. In a place where dengue is widespread, people tend to be complacent and unable to optimise the prevention measures. This is in line with a study in India, where dengue is endemic and the risk of dengue transmission can be averted by strong preventive measures that are dependent on both perceived susceptibility [36] and a person’s knowledge of the disease. Similarly, a strong association between dengue prevention behaviour and perceived high susceptibility has been observed in Malang, Indonesia [37].

Another latent factor is the perceived barrier to dengue infection. This barrier can be defined as the feeling that there are obstacles to performing recommended health behaviours. This inability to take action could stem from intrinsic factors, such as low motivation and the perception of a low risk of infection [38]. External causes such as low community engagement and participation can further contribute to this barrier [39, 40]. These problems are not solely experienced in this country; similar issues have been experienced related to dengue elsewhere [41, 42]. Perceived barriers should therefore be included in the domain of risk perception.

The latent factor of perceived severity refers to a person’s feelings about the seriousness of contracting a disease, or of not seeking treatment. Specific to item D4, the respondents were assessed on treatment-seeking behaviour that indirectly reflects how likely they are to be tested for dengue. Meanwhile, item D3 challenges the idea of death due to dengue. This measure of severity was highlighted in the previous literature, demonstrating that people perceived dengue infection to be more severe than Zika infection, even though both diseases are transmitted by the same vector [43]. They are therefore extra careful to avoid bites from mosquitoes possibly carrying dengue. This component of HBM has a profound effect on the course of other communicable diseases in this country [44]. Perceived benefit was the last latent factor in the risk perception domain; this is the individual’s perception of actions intended to reduce the threat of disease. Studies have shown that people are likely to adopt a behaviour change when their actions produce a positive impact [45]. Spending more money, as well as taking part in a dengue campaign (item D9 and D7), would certainly bring health advantages. Hence, the inclusion of these attributes in the RPAP questionnaire adds more value to the risk perception measurement.

The attitude domain is comprised of two latent factors: 1) cues to take action, and 2) self-efficacy. These are also components of the HBM. Cues to take action are motivations needed to trigger behaviours; self-efficacy is defined as the confidence in the ability to successfully perform a behaviour, during the decision-making process of whether to accept a recommended health action. This domain allows us to measure its association with risk perception. A person with a high perceived risk of dengue infection may have a positive attitude towards dengue prevention, as reflected by the HBM and as reported in various literature. For instance, an earlier local study found a positive correlation between high risk of infection and attitudes towards dengue prevention activity [46].

The final domain of the RPAP questionnaire, practice, includes two latent factors that measure good dengue prevention practice: 1) behaviours inside the house, and 2) behaviours outside the home or neighbourhood. Given *Aedes* sp. mosquitoes’ ability to breed easily in a human environment, it is important to ensure that both living spaces and surrounding areas are unlikely to become the breeding source. This work requires total participation by the respondents to keep the place safe. This domain also included several questions specifically about community involvement programmes (i.e., communal work or *gotong royong*), which have been reported to halt dengue transmission in certain areas [47]. In Malaysia, this community participation has its own platform called COMBI (Communication for Behavioural Impact) that plays a pivotal role in dengue prevention, especially in terms of an eco-bio-social approach [48]. COMBI is an important measure because a sustainable dengue prevention program will depend on the continuous commitment of the population [49].

The newly developed RPAP questionnaire has three main strengths. First, the content of the new instrument is backed by scrupulous research, as evidenced by the comprehensive literature review and structured guidelines, gathering of expert opinions, ample time for cognitive debriefing interviews, and pretesting phase. Secondly, the concurrent use of dual statistical approaches helps to complement the analysis and generate especially powerful results. Finally, the fact that the questionnaire is bilingual helps to reduce possible information bias and comprehension issues among multi-ethnicity Malaysian respondents with different levels of national language proficiency [50, 51]. However, the study’s greatest limitation is the online recruitment of the sample respondents. The population that was available as samples were confined to groups with a computer or mobile device, internet connection, and the ability to access a web survey. This method excludes socioeconomically disadvantaged populations, likely those experiencing health inequality. In addition, the RPAP questionnaire was not subjected to a concurrent validity test. This lack was due to how few risk perception questionnaires there are currently available that are tailored to the local culture of this analysis; however, other domains are commonly assessed and reported. In that view, this study performed a double advanced statistical analysis to ensure its validity.

## Conclusion

The RPAP questionnaire was developed in accordance with the Health Belief Model theory and established infectious disease guidelines; it has been demonstrated to be valid through extensive dual statistical approaches. The RPAP questionnaire is suitable for use in population-based studies and dengue prevention efforts. Ultimately, this questionnaire can quantify individuals’ perceived risk of dengue infection, and this data can then used by authorities to strategically develop a sustainable public health intervention programme.

## Supporting information

S1 Data (XLSX)

S1 Questionnaire (DOCX)
