# Supplementary material for: A unique double tango: Construct validation and reliability analysis of risk perception, attitude and practice (RPAP) questionnaire on dengue infection
Source: PLoS One. 2021 Aug 24;16(8):e0256636. doi: 10.1371/journal.pone.0256636 (PMC8384171; doi:10.1371/journal.pone.0256636)
Supplement: S1 Questionnaire — (DOCX) [file pone.0256636.s002.docx]

**Risk Perception, Attitude and Practice (RPAP) on Dengue Infection**

| **Risk Perception Dengue Fever** | | ***Circle your answer)*** | | | | | | | |
| --- | --- | --- | --- | --- | --- | --- | --- | --- | --- |
| 1. | I am at risk to get dengue fever. | **1** | **2** | **3** | **4** | **5** | **6** | **7** | **8** |
|  |  | Strongly  Disagree | | | | Strongly  Agree | | | |
| 2. | Dengue fever is a seasonal disease, I will be safe from it if the dengue season has passed. | **1** | **2** | **3** | **4** | **5** | **6** | **7** | **8** |
|  |  | Strongly  Disagree | | | | Strongly  Agree | | | |
| 3. | I am bitten by mosquitoes every day, but I have never been infected with dengue fever. So, I am not at risk of getting dengue fever. | **1** | **2** | **3** | **4** | **5** | **6** | **7** | **8** |
|  |  | Strongly  Disagree | | | | Strongly  Agree | | | |
| 4. | Dengue fever can cause death. | **1** | **2** | **3** | **4** | **5** | **6** | **7** | **8** |
|  |  | Strongly  Disagree | | | | Strongly  Agree | | | |
| 5. | Fever for 3 days is worrisome to me. I feel that I cannot wait up to 5 days to get treatment. | **1** | **2** | **3** | **4** | **5** | **6** | **7** | **8** |
|  |  | Strongly  Disagree | | | | Strongly  Agree | | | |
| 6. | I have many close friends who have recovered from dengue fever, but I am still afraid of dengue. | **1** | **2** | **3** | **4** | **5** | **6** | **7** | **8** |
|  |  | Strongly  Disagree | | | | Strongly  Agree | | | |
| 7. | All the time and money I spent to stop dengue is worthwhile because I'm concerned about living a healthier lifestyle. | **1** | **2** | **3** | **4** | **5** | **6** | **7** | **8** |
|  |  | Strongly  Disagree | | | | Strongly  Agree | | | |
| 8. | It is necessary for me to ensure there are no breeding spots around my house. | **1** | **2** | **3** | **4** | **5** | **6** | **7** | **8** |
|  |  | Strongly  Disagree | | | | Strongly  Agree | | | |
| 9. | I need to be involved in every health campaign aimed to destroy mosquito breeding place, as it helps reduce the risk of dengue to my family. | **1** | **2** | **3** | **4** | **5** | **6** | **7** | **8** |
|  |  | Strongly  Disagree | | | | Strongly  Agree | | | |
| 10. | I need a lot of money to implement dengue prevention at home. | **1** | **2** | **3** | **4** | **5** | **6** | **7** | **8** |
|  |  | Strongly  Disagree | | | | Strongly  Agree | | | |
| 11. | I am very busy until I have no time to implement dengue prevention at home. | **1** | **2** | **3** | **4** | **5** | **6** | **7** | **8** |
|  |  | Strongly  Disagree | | | | Strongly  Agree | | | |
| 12. | I need to spend the weekend with my family rather than participating in gotong royong to prevent dengue. | **1** | **2** | **3** | **4** | **5** | **6** | **7** | **8** |
|  |  | Strongly  Disagree | | | | Strongly  Agree | | | |
| **Attitude on Dengue Prevention** | | **Scale** | | | | | | | |
| 13. | With at least one person who is knowledgeable about the disease in the house, he/she can help prevent the disease in the home. | **1** | **2** | **3** | **4** | **5** | **6** | **7** | **8** |
|  |  | Strongly  Disagree | | | | Strongly  Agree | | | |
| 14. | It is necessary for me to deliver information about dengue fever to my family members. | **1** | **2** | **3** | **4** | **5** | **6** | **7** | **8** |
|  |  | Strongly  Disagree | | | | Strongly  Agree | | | |
| 15. | I become more interested to take part in control/prevention of dengue when there are cooperation within the neighbourhoods. | **1** | **2** | **3** | **4** | **5** | **6** | **7** | **8** |
|  |  | Strongly  Disagree | | | | Strongly  Agree | | | |
| 16. | It is necessary for me to ensure old and unused items that can store water, are kept closed. | **1** | **2** | **3** | **4** | **5** | **6** | **7** | **8** |
|  |  | Strongly  Disagree | | | | Strongly  Agree | | | |
| 17. | It is necessary for me to ensure that the drainage or water flow system in my house to be properly maintained. | **1** | **2** | **3** | **4** | **5** | **6** | **7** | **8** |
|  |  | Strongly  Disagree | | | | Strongly  Agree | | | |
| 18. | I only to dispose rubbish at the designated place. | **1** | **2** | **3** | **4** | **5** | **6** | **7** | **8** |
|  |  | Strongly  Disagree | | | | Strongly  Agree | | | |
| 19. | I do not keep unused items that can store water. | **1** | **2** | **3** | **4** | **5** | **6** | **7** | **8** |
|  |  | Strongly  Disagree | | | | Strongly  Agree | | | |
| **Dengue Prevention** | | **Scale** | | | | | | | |
| 20. | I use mosquito repellent (lotion/spray/coil). | **1** | **2** | **3** | **4** | **5** | **6** | **7** | **8** |
|  |  | Strongly  Disagree | | | | Strongly  Agree | | | |
| 21. | I always keep water containers in my house tightly closed. | **1** | **2** | **3** | **4** | **5** | **6** | **7** | **8** |
|  |  | Strongly  Disagree | | | | Strongly  Agree | | | |
| 22. | I put larvicide into the water storage to kill the mosquito larvae. | **1** | **2** | **3** | **4** | **5** | **6** | **7** | **8** |
|  |  | Strongly  Disagree | | | | Strongly  Agree | | | |
| 23. | I keep my drainage system properly maintained. | **1** | **2** | **3** | **4** | **5** | **6** | **7** | **8** |
|  |  | Strongly  Disagree | | | | Strongly  Agree | | | |
| 24. | I made complaint to the authority when I found an illegal dumping site. | **1** | **2** | **3** | **4** | **5** | **6** | **7** | **8** |
|  |  | Strongly  Disagree | | | | Strongly  Agree | | | |
| 25. | Abandoned and damaged vehicles in the neighbourhood trigger my intention to take the necessary action. | **1** | **2** | **3** | **4** | **5** | **6** | **7** | **8** |
|  |  | Strongly  Disagree | | | | Strongly  Agree | | | |
| 26. | I made complaint to the authority when there is damaged vehicle idling in my neighbourhood. | **1** | **2** | **3** | **4** | **5** | **6** | **7** | **8** |
|  |  | Strongly  Disagree | | | | Strongly  Agree | | | |
| 27. | I check for potential mosquito breeding place around the neighbourhood. | **1** | **2** | **3** | **4** | **5** | **6** | **7** | **8** |
|  |  | Strongly  Disagree | | | | Strongly  Agree | | | |
| 28. | I made complaint to the authority when I found illegal garden. | **1** | **2** | **3** | **4** | **5** | **6** | **7** | **8** |
|  |  | Strongly  Disagree | | | | Strongly  Agree | | | |
| 29. | I made complaint to the authority when I found illegal building structure. | **1** | **2** | **3** | **4** | **5** | **6** | **7** | **8** |
|  |  | Strongly  Disagree | | | | Strongly  Agree | | | |
